# Gaze movements and spatial working memory in collision avoidance: a traffic intersection task

**DOI:** 10.3389/fnbeh.2013.00062

**Published:** 2013-06-06

**Authors:** Gregor Hardiess, Sabrina Hansmann-Roth, Hanspeter A. Mallot

**Affiliations:** Cognitive Neuroscience, Department of Biology, Institute of Neurobiology, University of TübingenTübingen, Germany

**Keywords:** traffic intersection task, gaze movements, collision avoidance, potential-of-collision, spatial working memory, homonymous hemianopia, visual impairment, field loss compensation

## Abstract

Street crossing under traffic is an everyday activity including collision detection as well as avoidance of objects in the path of motion. Such tasks demand extraction and representation of spatio-temporal information about relevant obstacles in an optimized format. Relevant task information is extracted visually by the use of gaze movements and represented in spatial working memory. In a virtual reality traffic intersection task, subjects are confronted with a two-lane intersection where cars are appearing with different frequencies, corresponding to high and low traffic densities. Under free observation and exploration of the scenery (using unrestricted eye and head movements) the overall task for the subjects was to predict the potential-of-collision (POC) of the cars or to adjust an adequate driving speed in order to cross the intersection without collision (i.e., to find the free space for crossing). In a series of experiments, gaze movement parameters, task performance, and the representation of car positions within working memory at distinct time points were assessed in normal subjects as well as in neurological patients suffering from homonymous hemianopia. In the following, we review the findings of these experiments together with other studies and provide a new perspective of the role of gaze behavior and spatial memory in collision detection and avoidance, focusing on the following questions: (1) which sensory variables can be identified supporting adequate collision detection? (2) How do gaze movements and working memory contribute to collision avoidance when multiple moving objects are present and (3) how do they correlate with task performance? (4) How do patients with homonymous visual field defects (HVFDs) use gaze movements and working memory to compensate for visual field loss? In conclusion, we extend the theory of collision detection and avoidance in the case of multiple moving objects and provide a new perspective on the combined operation of external (bottom-up) and internal (top-down) cues in a traffic intersection task.

## Introduction

### Collision avoidance

Successful and efficient motion in space is based on estimates of the pose and motion of the own body as well as on estimates of (relative) position and independent motion of environmental objects. Motion plans need not only be suited to reach a goal at a given position in space, but at the same time need to take into account both stationary and moving obstacles. Avoidance of moving obstacles requires some sort of anticipation of the obstacles' movement, which may be expected to follow the rules of kinematics as well as traffic regulations if the obstacles are cars on an intersecting street or pedestrians on a sidewalk. The mental representation necessary for path planning and obstacle avoidance in a dynamically changing environment has been described as the *field of save travel* by Gibson and Crooks (1938). This safe field represents all the paths that an agent may take which will avoid collisions with objects in the traffic environment. The field is dynamic, i.e., it changes as the agent or other objects move. Its built-up and maintenance during spatial maneuvers require attentional processes controlling the intake and selection of information and the update of environmental models.

### Bottom-up processing and mechanisms for interceptive actions

When planning a trajectory in a cluttered dynamic environment, the navigator needs to assess the potential of independently moving objects to collide. We will call this variable the potential-of-collision (POC). The main source of information for judgments of POC is the optical (or retinal) flow (see, for example, Lappe et al., 1999; Fajen, 2005) and in particular the perception of time-to-contact (Lee, 1976). The time passed between visual removal and collision or near contact with an object is also known as time-to-collision (Brown and McFaddon, 1986), time-to-arrival (Schiff and Oldak, 1990), or time-to-passage (Kaiser and Mowafy, 1993). Originally, the concept of time-to-contact was introduced into the literature as a hypothesis of how behavior involving interactions with moving objects (such as catching or hitting a ball, i.e., interceptive actions) could be timed (Lee, 1980). An object's time-to-collision can be calculated as the ratio of the object's image size to the rate of change of this size; this ratio was termed *tau* by Lee (1976). The information provided by *tau* is sufficient for the timing of interceptive actions, avoidance maneuvers, and psychophysical judgments of time-to-collision (e.g., Wagner, 1982; Lee et al., 1983; Savelsbergh et al., 1991, 1993). However, despite the fact that *tau* requires just simple computations, only few studies have presented direct neuro-scientific evidence for the usage of *tau* (see Wang and Frost, 1992; Sun and Frost, 1998; Frost, 2010). In fact, empirical results and formal analyses have accumulated which suggest that the *tau*-hypothesis may not be valid and alternative approaches have been put forward (for review see: Wann, 1996; Tresilian, 1999), i.e., *tau* is limited by several factors. For instance, estimates of *tau* are highly sensitive to noise and require an object that is spherically symmetric. Further, *tau* may be used to specify the time-to-contact, but is does not allow to detect changes in velocity. For this task, the first-order temporal derivative of *tau* (i.e., rate of change), *tau*-*dot*, has been shown to contain the necessary information (Coull et al., 2008). However, evidence for the use of a constant *tau*-*dot* strategy is conflicting and not convincing (e.g., Bootsma and Craig, 2003 or Yilmaz and Warren, 1995). Furthermore, experiments show that *tau*-*dot* is just perceived passively from changing visual parameters and estimates based on it have limited predictive power. Festl et al. (2012) studied the related quantity *rho*, i.e., the rate of velocity change, which can also be extracted from retinal measurements alone. Again, experimental results do not support the use of this quantity in self-motion estimation. Recently, Keil and López-Moliner (2012) proposed a new neuro-physiologically plausible implementation based on *tau*, i.e., the corrected and modified *tau*-function. In an initial step, a modified *tau*-function was suggested capable to describe the neuronal responses of object approaches (with constant velocity) found in different species (locust, fruit fly, bullfrog, and pigeon). Furthermore, a corrected *tau*-function was formulated to estimate time-to-collision for “sufficiently small” angular sizes, which, as compared to *tau*, has the advantage of being less sensitive to noise. Finally, the authors showed that the new framework accounted well for the performance of psychophysical experiments where subjects had to estimate time-to-collision of a single, linearly approaching object. However, despite providing a neuro-physiologically plausible implementation of *tau*-based functions, the predictive power in the more general context of timing interceptive actions as well as collision avoidance remains to be shown in future studies.

Besides image expansion or looming, the source of information used for *tau* and *tau*-related functions, the angular direction of an object (i.e., object bearing) also contains information for detecting a collision event. Object bearing (i.e., the angle between the heading and an intercepting object) can be measured over a range of time. Objects with high POC will have bearing shifts, which are close to zero (constant bearing angle strategy; Lenoir et al., 1999). In detecting collisions, both sources of information might be involved determining that a particular object is expanding and that the angular direction is constant (e.g., Andersen and Kim, 2001; Ni and Andersen, 2008).

In order to deal with collision detection and avoidance a number of potential variables (where *tau* is just one of them) have been identified which are available in the sensory input and can in principle be used to obtain an estimate of POC which is adequate to solve the collision task [e.g., depth perception, Gray and Regan (1998) and Cavallo and Laurent (1988); size and motion based information, DeLucia and Warren (1994); see also review Zago et al. (2009)]. However, it is much less clear on what visual information subjects do actually rely in order to avoid collisions with objects in the path of motion and how this information is selected when various sources are present? Here, studies are needed indicating the relevant environmental cues and the mechanisms of selecting these cues from the sensory input. Suitable attempts in understanding such search and identification behavior will include the analysis of gaze movements associated with detection of relevant visual stimuli and directing attention. Overall, it seems clear that gaze does play an important role relevant in obstacle since more than 90% of traffic accidents are due to problems with the acquisition of visual information (e.g., Sivak, 1996). To date just a few studies are published analyzing attentional processes in multiple-object collision detection (Andersen and Kim, 2001; Vaux et al., 2010). Additionally, less attention was given to gaze movements in service of gathering relevant information from the multiple collision events. In the present paper, we provide studies which combine the presentation of multiple moving (and collision relevant) objects with the measurement of gaze movements in a traffic intersection task.

## Hypotheses about the role of gaze and spatial memory in collision avoidance

As mentioned before, the main emphasis in studies of obstacle avoidance behavior has been on sensory cues (e.g., retinal flow patterns) and their bottom-up processing. With bottom-up processing visuo-motor control can proceed without awareness (pre-attentively), which is probably the normal mode regarding fast and skilled interceptive actions, i.e., interceptive actions bypass cognitive operations. More recently, the key role of prior knowledge (i.e., priors related to temporal and spatial characteristics of the physical world) and internal models (i.e., top-down processing) associated with the allocation of attention for guiding interceptive behavior has attracted increased interest (Land and McLeod, 2000; Andersen and Kim, 2001; Wickens et al., 2001; Zago et al., 2004; Dessing et al., 2005; Jovancevic et al., 2006; López-Moliner et al., 2007; Mrotek and Soechting, 2007; López-Moliner and Keil, 2012; Diaz et al., 2013). Collision avoidance in dynamic environments involves the scanning of many potentially relevant obstacles, only a few of which are selected for tracking. It seems plausible that this process is based on three components; information intake by sensory processes, motion planning, and risk anticipation in a working memory stage, and the interaction of these two in attention and sensory-motor control. In this article, we will address these components.

In a series of experiments, we tested the following hypotheses and predictions: (1) Gaze patterns are adapted to the task of collision avoidance but do not predict the performance of individual subjects or trials (section Gaze Behavior in the Traffic Intersection Task). (2) The representation of cars within spatial working memory is task specific, i.e., cars with high POC are remembered better (ranked higher) than cars with low POC (section Allocation of Spatial Working Memory). (3) We predict that working memory processes are crucial for selecting cars with the most significant POC in street crossing. Consequently, subjects with working memory disorders should fail in collision avoidance. To test this hypothesis we investigated gaze patterns and collision avoidance in stroke patients showing similar visual field defects but differ in working memory function (section Role of Gaze and Working Memory in Visually Impaired Subjects).

## Experiments about collision avoidance

### Experimental paradigm—the traffic intersection task

**Figure 1:**
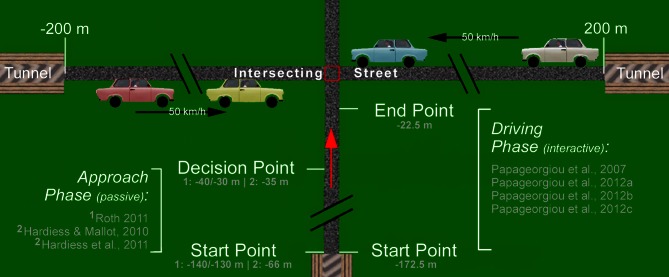
**Overview of the traffic intersection experiments used to analyze the function of gaze and visuo-spatial memory in the task of collision prediction or avoidance in normal and visually impaired subjects.** The overall structure of the intersection task used in all our experiments was as follows: beginning from a start point, subjects approached (passively or interactively, i.e., controlling their speed) the intersection while visually observing of the traffic on the intersecting street in order to predict or avoid a collision. The cars on the intersecting street had different colors and were uniformly distributed over two lanes (right-hand traffic); their number could be varied to allow for different traffic densities. The speed of the traffic cars was always constant with 50 km/h and their travel paths started and ended in tunnels. In passive trials, subjects approached until a decision point where estimates about a collision or the positions of traffic cars were made. In interactive trials, subjects were allowed to adjust their own driving speed (within certain limits) within the approach section between the start and end points marked in the figure. From there on, movement was extrapolated with constant speed and the occurrence of collisions was recorded.

**Figure 2:**
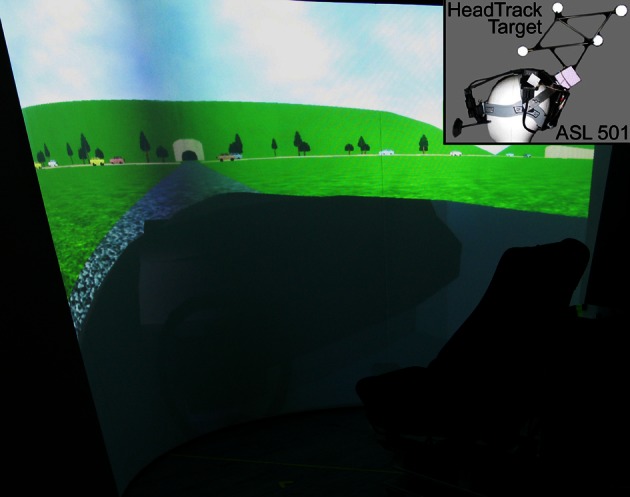
**Large-field projection screen (presenting the approach phase of the experiment) and seat used in the experiments.** The screen provides a large field of view of 150 by 70° in a seated but otherwise unrestricted subject. Inset: eye and head tracking devices with 60Hz sampling frequency (head: ARTtrack/DTrack from A.R.T., eye: model 501 from Applied Science Laboratories).

This section summarizes a series of experiments investigating gaze scanning strategies and their interactions with working memory for the purpose of obstacle detection and avoidance in a dynamic environment. To test subjects in a “real-world” scenario under controllable conditions, a virtual intersection task was developed where visually impaired as well as healthy subjects were required to detect and avoid collisions while approaching and crossing a street with two-directional traffic (see Figure 1). A driving simulator was used for all experiments (Figure 2). Eye and head tracking was combined to a gaze vector comprising azimuth and elevation of joint head and eye directions in a lab-based frame of reference. The approach to the intersection was along a straight road with prescribed or self-controlled speed, but with no additional traffic, traffic signs, or curves. Thus sensory motor tasks such as steering, checking the rear-view mirror, looking out for road signs, other vehicles, or pedestrians, as well as gear shifting were excluded. Attentional actions are therefore restricted to monitoring the crossing traffic, including the selection and monitoring of cars considered as possibly threatening a collision. Such cars are relevant for the task of adjusting the own velocity in order to hit a gap in the crossing traffic. Thus, by presenting two streams of potentially hazardous cars (rather than just one intercepting object) we extend the task of time-to-collision estimation to the process of sampling and selecting collision relevant items. Consequently, subjects are required to constantly engage in two processes, first, sampling and representing the traffic scene in an adequate way (i.e., in terms of task relevance) and second, calculating and predicting POC for selected cars in order to generate appropriate behavior.

### Role of gaze and spatial memory in subjects with normal vision

#### Gaze behavior in the traffic intersection task

A non-interactive version of the traffic intersection task was used to investigate the role of gaze movements in collision avoidance (Roth, 2011). Specifically, we addressed the question whether individual gaze movements or more complex gaze patterns are predictive for task performance (i.e., collision detection). In a variety of comparable trials, 25 healthy subjects were passively watching an approach to the intersection (see Figures 1, 2) with one of eight traffic configurations (four of which would entail a collision). Before reaching the crossing, the display stopped and all cars were hidden. The subjects then had to decide if they would pass the crossing without a collision or not.

Traffic situations were derived from two basic arrangements with a total of 19 cars driving in both directions. One of these cars would cause a collision if the approach would have been continued to the intersection. To avoid learning effects while repeatedly confronting a subject with one and the same traffic situation, the two traffic situations were mirrored and car colors were changed in a random fashion in each trial. This results in four traffic scenarios with a collision relevant car (i.e., hit or miss trials) and four additional collision-free conditions (i.e., correct rejection or false alarm trials) generated by simply removing the relevant car. All traffic cars had the same constant speed of 50 km/h. During the approach phase (length: 100 m; duration: 9 s), subjects passively drove toward the intersection with a constant speed of 40 km/h (Figures 1, 2). Two different start and decision points (140/40 m and 130/30 m in front of intersection) were assigned randomly to the trials. At the decision point, the remaining distance to the intersection was therefore 40 or 30 m, respectively. Each subject performed 80 trials, 40 with a collision relevant car and 40 without, in randomized order. At the decision point all cars were hidden (time-to-collision of the collision relevant car at the time of hiding was 3.6 or 2.7 s, respectively) and the subject was ask to answer the question “would you cause a collision assuming that the approach proceeds with the same speed?” with “yes” or “no.” After answering the question a feedback (crash “yes” or “no”) was given to the subject.

**Figure 3:**
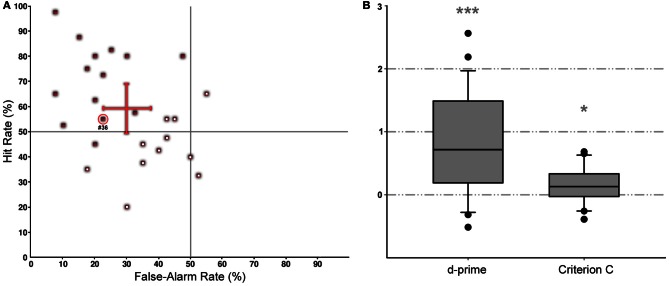
**Collision detection performance. (A)** Distribution of individual hit and false alarm rates shown for all 25 subjects (red squares). Data points significantly different from zero are marked by open symbols. Values for the mean and standard deviation of the hit (value: 59 ± 20%) and the false alarm rates (value: 30 ± 14%) are depicted as a red cross. The red circle denotes that subject (#36) for which scan-path details are shown in Figure 4. **(B)** Box plots of the averaged discriminability index (d-prime; left) and the averaged criterion for an observer bias (C; right). Statistical effects (one-sample *T*-Test, test value: zero) are presented for criterion C and d-prime (^*^p < 0.05; ^***^p < 0.001).

The overall task performance (i.e., percentage of correct judgments of the subjects) was high, i.e., the average of d-prime values [*d*′ = *z*(Hit) − *z*(FalseAlarm); Swets, 1996] was significantly above zero (Figures 3A,B). Hit rate per subject ranged from 20 to 100% while false-alarm rate varied only between 5 and 55%; only two subjects showed false-alarm rates above 50% for no-crash trials (Figure 3A). D-prime values were significantly above zero (*P* < 0.05) according to Marascuilo's one-signal test (Marascuilo, 1970) for 14 out of 25 subjects. Subjects' performance differs with respect to both, d-prime and criterion values. The criterion *C* was calculated to detect any observer bias (*C* = −0.5 × [*z*(Hit) + *z*(FalseAlarm)]). The averaged bias (Figure 3B) was slightly positive indicating a tendency toward “no” responses (i.e., a more conservative performance—in case of doubt subjects will rather say that no crash will occur). Note that an undetected crash resulted simply in the presentation of a “crash sign” with no further consequences for the subject.

**Figure 4:**
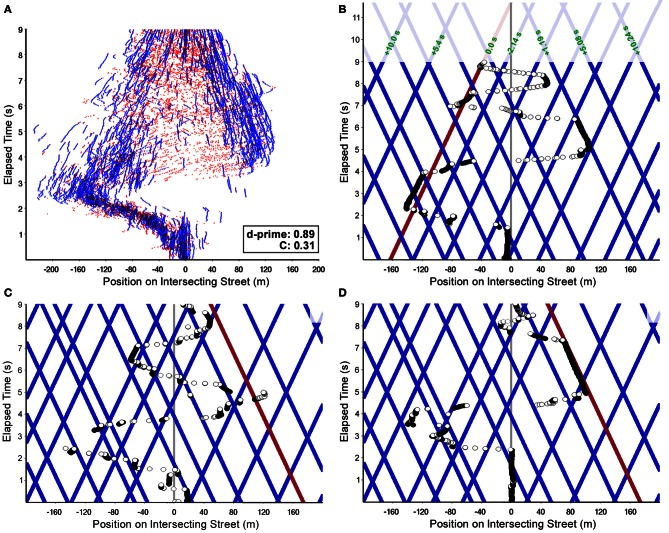
**Scan-path examples of a representative subject (#36; hit rate: 55%, false-alarm rate: 22.5%; see Figure 3A). (A)** Gaze scan-paths for all 80 trials performed by the subject are plotted together. Blue parts of the scan-paths denote fixations and red ones saccades. The left-right-left pattern of gaze movement combined with local scanning was found as general pattern in all subjects. Note the inward slant of the blue segments, indicating that object fixations almost exclusively landed on to cars approaching the intersection (d-prime and observer bias are reported for this subject). **(B)** Exemplary scan-path of the approach to the intersection for a single hit trial. The positions of all cars over time are shown in blue; the collision relevant car is depicted in red, i.e., the red line hits the subjects' trajectory (vertical midline) at time 11.7 s. Position lines of all cars after the time the cars disappear are drawn with transparency. Fixational elements of the gaze scan-path are drawn as black, positions during saccades as white circles. Differences in time-to-collision for all cars with relevant POC are given relative to the colliding one (numeric values in green). **(C)** Another exemplary scan-path of the approach to the intersection for a hit trial where no relevant fixations to the colliding car occur. **(D)** Exemplary scan-path of the approach to the intersection for a single miss trial. Please note, despite fixating the collision relevant car for about 2 s, the subject failed to detect the high POC of that car.

The collision detection task requires the assessment of the POC of 19 (initial number, cf. Figure 4B) individual cars appearing at the onset of the experiment. Possible strategies for selection, scanning, and POC estimation can be inferred from the subjects' gaze patterns performed during the 9 s approach phase. The overall gaze pattern we found was determined by an initial shift to the left followed by a shift to the right side within the first 6 s (cf. Figure 4). During the last 3 s, additional left-right oscillations are noticeable. The initial left-right-left pattern occurring in most of our subjects was assumingly due to a well-trained and habituated gaze behavior on streets with right-hand traffic. Besides this global gaze pattern, local oscillations also occurred allowing to sample and select the most relevant cars regarding their POC (Figure 4).

**Figure 5:**
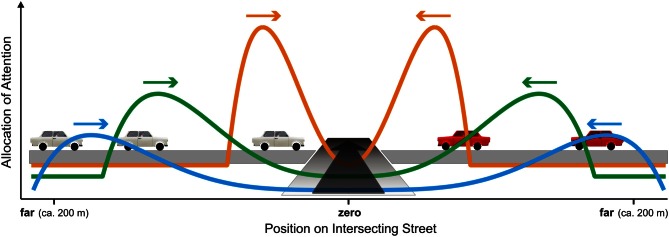
**Schematic account of the allocation of overt attention (i.e., distribution of fixations) during the approach to the intersection is scaled by the subject's distance to the intersection (blue: furthest, green: intermediate, and orange: closest) combined with the position of the traffic cars.** Additionally, only cars moving toward the intersection are considered (arrows).

In addition to this global scanning behavior, two more task-specific properties of gaze behavior can be observed. First, only cars moving toward the intersection (“inwards”) are taken into consideration while cars that have already passed the intersection point are ignored. This can be seen from Figure 4, where almost all fixational segments of the scan-paths are slanted inwards. That is, although cars driving in both directions were visible at each position along the intersecting street, subjects clearly select the cars with larger POC. Second, in terms of eccentricity, cars with high POC will occur around positions *x*_*c*_ = *z* × *v*_*c*_/*v*_*s*_, where *x*_*c*_ is the distance of the car from the intersection, *z* the subject's distance from the intersection, and *v*_*c*_, *v*_*s*_ are the velocities of the intercepting cars and the subject, respectively. In Figure 4, traces of cars approximately satisfying this condition are shown in red. As is best visible from Figure 4A, the distribution of fixation targets clearly peaks around the visual direction with highest POC. Taken together, both selection mechanisms generate a spatio-temporal pattern of the allocation of attention summarized in Figure 5.

The overall attentional resource which must be allocated adequately to the traffic cars is thought to be fixed and limited (i.e., only about four items can be simultaneously represented in working memory, e.g., Brady et al., 2011). Furthermore, we assume that subjects shifted their attention in an overt fashion, i.e., shifts of attention are associated with gaze movements [cf. shift of attention toward foveal vision in complex traffic stations: Miura (1987) and Crundall (2005)]. At the beginning of the approach, a substantial amount of attention should be allocated to cars in the far periphery of the intersection (Figure 5, blue curve). In this early stage, attention can be allocated broadly and with low amplitude (i.e., intensity) because the actual POC is relatively small as long as the intersection is distant. During the approach, the attention is zooming in to the margins of the intersection and ends up as a localized, high amplitude peak (Figure 5, orange curve). Here, the relevant time window is very small and POC considerations concern only one single position of possible colliding cars.

Figures 4B,C shows a typical scan-path of a single approach for a crash trial (colliding car in red). The overall scanning is shaped by the global left-right-left pattern (cf. also Figures 4A,D). Additionally, a pattern of shifting attention overtly as illustrated in Figure 5 is obvious. During scanning, fixations with short duration are applied to cars in order to sample for their possible POC (selection). Long lasting fixations are directed to selected cars (two cars in Figure 4B), presumably to estimate their actual POC. Such estimates of POC can be obtained by two strategies (i.e., image-based and memory-based). In an image-based approach, image-expansion (processes based on *tau*; see section Bottom-Up Processing and Mechanisms for Interceptive Actions) as well as object bearing are processes that might be involved in estimating the POC (cf. Andersen and Kim, 2001). Object bearing [i.e., the angle between the heading and an intercepting car, tan^−1^(*v*_*c*_/*v*_*s*_)] can be measured over a range of time. Cars with high POC will have bearing shifts, which are close to zero (constant bearing angle strategy; see section Bottom-Up Processing and Mechanisms for Interceptive Actions). Here, bearing could be based on retinal (without gaze-shifts) or extra-retinal (gaze shifts are possible and object positions must be updated in a visual array) representations. In any case, the constant bearing strategy will work only if the observer and objects translate at constant velocities and along linear trajectories. Indeed, these requirements were met in our experiment. However, in a similar experiment with variable relative speed of object and observer, subjects applied similar and comparable gaze patterns (cf. Figure 8 and Papageorgiou et al., 2012c). In addition, Andersen and Kim (2001) found in collision detection experiments where multiple objects were present only small interference effects concerning the bearing angle. In conclusion, we cannot completely rule out the possibility of relying on an image-based representation. However, the exclusive use of such a format seems unlikely.

Dealing with multiple targets and keeping track of trajectories over time requires a working memory allowing to store the relevant parameters for each target. This storage might be a simple list of the sensory data obtained for each object, but allows to integrate incoming data with previously stored data of the same object, as well as extrapolation of future states. One could also think of this working memory as an ego-centric “mental” map featuring object position and trajectories, where the frame of reference is given in optic flow variables such as nearness (distance over ego-speed), bearing, etc. The raw data that this map is based on is the same as in the image-based case, the difference of the two approaches being the presence of a working memory component.

If working memory is involved, distance can also be judged from the image size of the cars and prior knowledge about car sizes. The estimation of distance based on familiar size has been found also for other tasks, such as the interception of free-falling objects (Tresilian, 1993; Peper et al., 1994; Wann, 1996). The phenomenon of speed constancy, where the speeds of two objects located at different distances but moving physically with the same speed, are perceived as equal, accounts also to this idea of relying on familiar size (Palmer, 1999; Distler et al., 2000). Note that an internal model of known size need not be considered as a cognitive, declarative parameter (López-Moliner et al., 2007). It may rather result from a process of calibration in which subjects learn, through experience, to adapt their internal representation.

The built-up of a working memory over time is likely related to the pattern of gaze movements. Repeated or prolonged fixation or tracking of a target will improve the accuracy of image-based measurements and therefore the working memory representation. In this sense, gaze movements may contribute to speed estimation in depth, vertical elevation, and horizontal azimuth (e.g., Brenner and Smeets, 2011; Spering et al., 2011). Smooth pursuit can be accurate to within 5% for target speeds up to about 57°/s (Bahill and McDonald, 1983). Bennett et al. (2007) found that subjects can estimate the acceleration of a temporarily occluded object by the use of smooth and saccadic eye movements. Our data indicate that subjects use many but brief fixations to gain an overview of the most relevant cars, while a small number of long lasting fixations are used for the POC calculation of the selected cars. We conclude that a working memory of the described type (i.e., some sort of local “mental map”) is involved in these processes.

**Figure 6:**
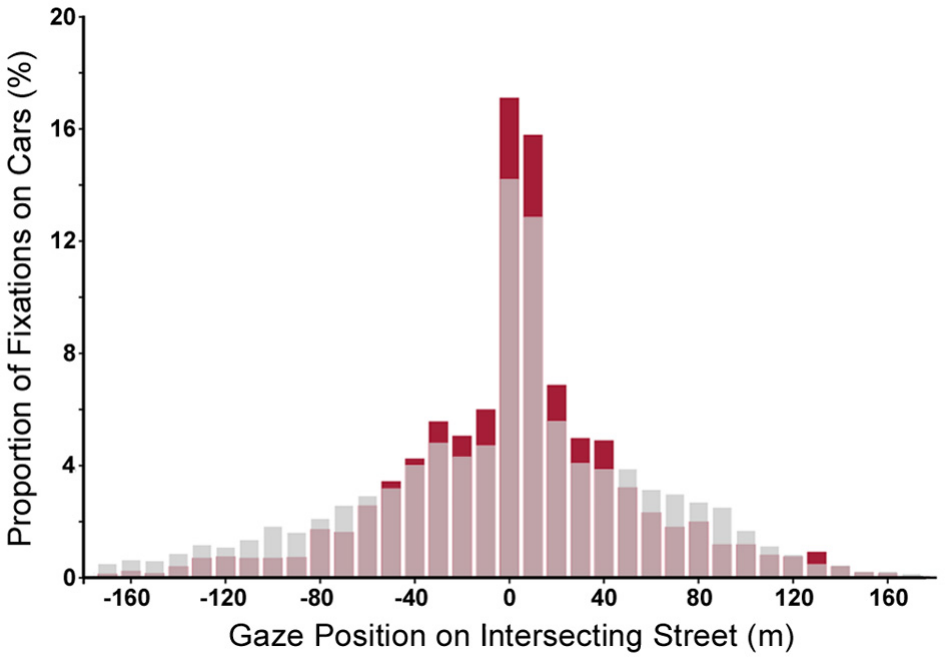
**Distribution of task relevant fixations applied in all crash trials shown as proportion of fixations on cars (during the approach) for good performers (subjects with hit rates above 50%, red bars) and for poor ones (subjects with miss rates above 50%, gray bars).** Redrawn from Roth (2011).

Subject's gaze behavior is clearly adapted to the visual presentation and the task. It therefore seems plausible to assume that a correlation exists between the gaze pattern in a given task and the performance. However, no such correlation was apparent from our experiments employing extensive analyses of relevant gaze parameters. As an example, Figure 6 shows the distribution of the landing points of all fixations for one traffic situation. The distribution for good performing subject is slightly more focused on the intersection point, but the differences are not large enough to allow a prediction of collision detection performance from gaze pattern.

One gaze parameter that seems to be slightly predictive of task performance is re-fixation rate. While overall, re-fixation rate for the collision relevant car is rather low (about 30%), subjects performed significantly more re-fixations on that car in hit trials as compared to miss trials. In addition, re-fixations were more likely to occur during the second half of the approach (from 4.5 to 9 s).

#### Allocation of spatial working memory

**Figure 7:**
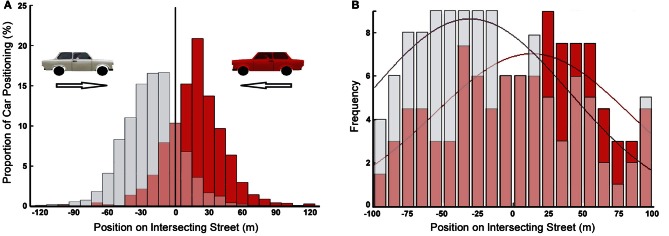
**Task specific representation of cars in working memory shaped by their POC (gray: cars moving from left to right; red: cars moving from right to left). (A)** Distribution of number of cars positioned on the intersection in the memory recall phase (i.e., reconstructing the traffic scene) of the traffic intersection task [redrawn from Hardiess and Mallot (2010)]. **(B)** Frequency of correct detection of change due to cars removed from all positions on the intersecting street (i.e., only hit trials are considered) in the change detection version of the traffic intersection task [redrawn from Hardiess et al. (2011)]. The smooth curves show normal distributions fitted to the data.

Independence between gaze behavior and task performance in collision avoidance was also reported by Hardiess and Mallot (2010). In this study, a modified version of the collision avoidance paradigm (Figure 1) was used to investigate how well each car is represented in working memory. Here, subjects passively approached the intersection up to a certain point where the approach stopped and all traffic cars disappeared. Then, subjects were required to reconstruct from memory the last traffic configuration as seen at the time of stopping. For the reconstruction task, images of cars heading left and right were presented in the upper part of the display screen. Subjects used a joystick to drag and drop these cars to the remembered positions on the intersecting street. The placement positions were recorded in each trial. Figure 7A shows summary results for the position and heading direction of placed cars. Cars driving rightwards are mostly placed left of the intersection while cars driving left are placed predominantly on the right side. Thus, cars with high POC are remembered better than cars with low POC. This may be due to selective information take-in as demonstrated in Figure 4, to selective memory formation in working memory, or to both of these effects.

Task specific working memory representation was also revealed in a change detection version of the traffic intersection task conducted by Hardiess et al. (2011). Here, during the approach subjects had to observe different traffic situations in preparation of collision avoidance. In a subset of trials, the approach stopped before reaching the intersection. After a short delay (330 ms) during which the screen was turned blank, a traffic configuration was displayed which either equaled the final arrangement at stopping, or differed by removal of individual cars at various positions along the intersecting street. Subjects were then requested to report recognized differences between the presented configuration and the configuration at the end of the (interrupted) approach phase (i.e., change detection task). Figure 7B shows the frequency of correct detections as a function of car position and driving direction. While the distributions are broader than in the previous experiment, the advantage of cars approaching the intersection (i.e., cars with high POC) is clearly apparent.

In conclusion, gaze patterns were not found to be predictive of task performance, i.e., we found almost the same gaze behavior regardless of whether collisions were predicted correctly or not (cf. also Figures 4B,D; similar gaze pattern despite different performance). However, during the processes of visual perception ending in the representation in spatial working memory cars were weighted based on their POC. These results together with the fact that subject's performance varies substantially, indicate that subjects must differ in the mental processes of representing the equally selected (by means of gaze) material within spatial memory. Such variations could be related to differences concerning executive functions, capability of working memory, or learning ability.

### Role of gaze and working memory in visually impaired subjects

In this section, we report findings about collision avoidance in patients with homonymous hemianopia regarding their ability to recruit gaze as well as memory resources to compensate the visual field loss. Hemianopic scanning is primarily visually elicited, i.e., patients with homonymous visual field defects (HVFDs) can be seen as a kind of model for visual exploration under low vision. In order to underline the role of active vision in the patients' compensational behavior, data about demographic and clinic factors are presented and shown to be not predictive of collision avoidance performance. Rather, functional gaze adaptation in combination with spared working memory capacities enables a subgroup of patients to adequately compensate their visual field loss.

#### Collision avoidance in persons with homonymous visual field defects (HVFDs)

Patients with HVFDs are impaired by a binocular restriction of the visual field caused by unilateral post-chiasmal brain damage due to cerebrovascular accident, traumatic brain injury, or tumors (Zihl, 1994, 2000; Kerkhoff, 1999). Depending on magnitude and site of the lesion, HVFDs can comprise small scotomas, loss of one visual quadrant, or larger losses affecting up an entire visual hemifield (half side loss). HVFDs create a marked amount of subjective inconvenience in everyday life (Papageorgiou et al., 2007; Gall et al., 2009). Patients with HVFDs may show impairments of reading, visual exploration and navigation, collide with people or objects on their blind side, and may be deemed unsafe to drive (Trauzettel-Klosinski and Reinhard, 1998; Zihl, 2000). This has led to the belief that homonymous visual field loss is *per se* associated with functional impairment.

Driving has been considered to be problematic for patients with HVFDs and investigations concerning the performance of hemianopics in realistic or simulated driving report a variety of findings. The majority of studies have highlighted poor steering control, incorrect lane position, and difficulty in gap judgment as the primary problems of drivers with HVFDs (Szlyk et al., 1993; Tant et al., 2002b; Bowers et al., 2009, 2010; Wood et al., 2009). Further, in an interactive version of the traffic intersection task, Papageorgiou et al. (2012a) studied one of the core abilities in driving—collision avoidance—in 30 patients with HVFDs (20 with hemianopia and 10 with quadrantanopia) and 30 normal-sighted group-age-matched controls. In a large number of trials, subjects had to actively (i.e., continuous adjustment of the own speed between 18 and 61 km/h by means of a joystick) hit a gap between cars moving on an intersecting street (cf. Figure 1). In two experimental conditions, the density of traffic cars was adjusted to achieve two different collision probabilities, i.e., 50 and 75%, respectively. When analyzing all patients together, subjects with HVFDs had on average more collisions than subjects with normal vision. The difference between the controls and patients was about one more crash for 50% density and two more crashes for 75% density. In density 75%, hemianopics experienced more collisions with vehicles approaching from the blind side than the seeing side. Additionally, in density 75%, the number of collisions on the seeing side of subjects with HVFDs was similar to the number of collisions experienced by normal subjects. In the easier task (density 50%), differences in collision rates between the blind and seeing hemifield were not obvious. These results suggest that patients with HVFDs were less efficient and experienced difficulties in collision avoidance when intersecting a traffic street. In a further analysis (Papageorgiou et al., 2012c) we subdivided the collective of HVFD patients based on the number of collisions caused in 50 and 75% density trials. Thus, the splitting involved a joined performance measure for the two tasks of different difficulty and resulted in two subgroups (adequate: HVFD_A_ and inadequate: HVFD_I_) each one consisting of 15 patients. HVFD_I_ patients still showed reduced collision avoidance as compared to healthy controls for both traffic densities. In contrast, the subgroup of HVFD_A_ patients showed performance values identical to controls for both densities and for crash number as well as trial duration. This result, i.e., that the adequate subgroup of patients performed similar to controls, was also shown for other visual tasks (Zihl, 1999; Machner et al., 2009; Hardiess et al., 2010) indicating that some patients compensate functionally for the visual impairments while others do not or not to a sufficient amount.

Concerning visual exploration and collision avoidance it has been a matter of debate whether driving performance of patients with longstanding HVFDs is primarily determined by visual field parameters (e.g., the extent of the visual field along the horizontal meridian, Johnson and Keltner, 1983; size of the area of sparing within the affected hemifield, Papageorgiou et al., 2007), or affected by additional factors, such as age of patients, side of brain injury, time span since lesion onset, and compensation by adapted gaze and memory strategies (Pambakian et al., 2000; Papageorgiou et al., 2007; Hardiess et al., 2010; Wood et al., 2011). Papageorgiou et al. (2007) investigated the correlations between visual field measures and the collision avoidance performance in the interactive traffic intersection task as well as a vision-targeted and health-related quality of life score (i.e., NEI-VFQ-25) in a large population of 33 HVFD patients. Interestingly, neither collision avoidance nor performance in everyday life was found to correlate significantly with the extent of visual field loss. In a follow-up investigation, side of brain injury and time span since lesion onset could be excluded as factors influencing collision avoidance performance (Papageorgiou et al., 2012a). Under the tested clinical and demographic variables, the age of the patients was the only variable correlated with task performance (the older the patients the higher the crash rate). However, an effect of age on performance was also found for the age-matched healthy controls. In conclusion, it can be argued that clinical and demographic factors play just a minor role in explaining the variability of hemianopics in driving tasks (as well as in tasks that demand visual functions to a lower extent; cf. Hardiess et al., 2010). Rather, functional compensation of the HVFDs by applying adequate eye and head movements in combination with intact spatial memory will help the patients to overcome their limitations and to reach task performance in the range of healthy subjects.

#### Role of gaze and working memory for visual field compensation in collision avoidance

Studies with patients suffering from binocular visual field deficits (i.e., HVFDs) are instrumental in assessing the gaze strategies and their adaptation to reduced information intake (i.e., hemianopic scanning is primarily visually elicited, namely by the visual defect and not by additional brain damage; Tant et al., 2002a) and maybe reduced processing capacities (Papageorgiou et al., 2012b). As compared to healthy subjects, patients' strategies may differ with respect to scan-path pattern and memory involvement, leading to various levels of functional compensation. In the following sub-section, we review evidence for the hypothesis that gaze adaptation is the primary compensatory mechanism in patients with HVFDs to achieve collision avoidance, but that in addition to gaze adaptation, the availability of working memory capacities must also be considered.

From studies investigating visual exploration, it is known that patients with HVFDs are able to behave adequately compared to healthy subjects by applying compensatory gaze movements. In general, when viewing simple horizontal line patterns or line drawing, hemianopics spend most of the time looking toward their blind hemifield in order to bring more of the visual scene into their seeing hemifield (Ishiai et al., 1987). Such displacement of the fixation point toward the hemianopic side was considered to be an efficient compensatory strategy and was observed in numerous other basic tasks, including dot-counting (Zihl, 1995; Tant et al., 2002a; Hardiess et al., 2010), viewing of natural and degraded images (Pambakian et al., 2000) and visual search (Hardiess et al., 2010). Papageorgiou et al. (2012c) investigated gaze compensation of hemianopic patients in a more demanding task, i.e., the interactive traffic intersection task under dynamic and time-constrained conditions. In this task, participants (14 HVFD patients and 19 controls) were confronted with 30 different traffic situations presented in two traffic densities. The task was to adjust own driving speed to avoid any collision with a traffic car, i.e., to hit a gap in the crossing traffic [for task details see section Collision Avoidance in Persons with Homonymous Visual Field Defects (HVFDs), study by Papageorgiou et al. (2012a), and Figure 1]. To introduce a time-constrained situation, participants were not allowed to stop their driving at any point in time. Regarding their collision avoidance performance we divided the collective of patients in two subgroups [five adequate: HVFD_A_ and nine inadequate: HVFD_I_ patients; cf. section Collision Avoidance in Persons with Homonymous Visual Field Defects (HVFDs)].

**Figure 8:**
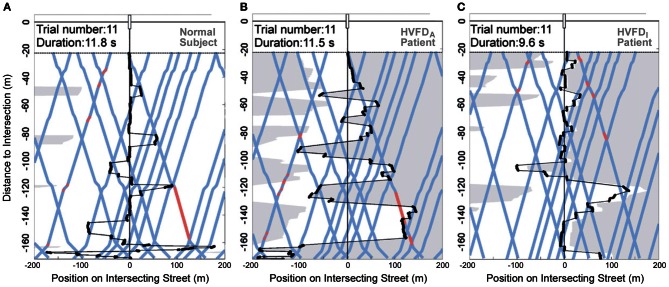
**Visualization of gaze trajectory.** Each plot demonstrates the participant's gaze pattern during approach to the intersection for a representative trial with 50% traffic density. A normal subject **(A)**, an adequate-HVFD_A_ patient with right hemianopia **(B)**, and an inadequate-HVFD_I_ patient with right hemianopia **(C)** are shown. Gaze position is depicted in black (fixations shown as circles and saccades as lines). Gray transparent areas are beyond the visual field boundaries. The blue lines represent the courses of the traffic cars and red segments indicate when vehicles are moving on collision course. Speed adjustments of the participant (acceleration or deceleration) result in kinks on the blue lines. The HVFD_A_ patient shows more gaze shifts, more fixations on cars, larger saccades, larger mean gaze eccentricity, and more speed adjustments (kinks) than the HVFD_I_ patient, who demonstrates decreased gaze activity. Trial duration is similar between the normal subject and the HVFD_A_ patient, while the HVFD_I_ patient completes trials in a shorter period of time. Redrawn from Papageorgiou et al. (2012c).

In order to identify gaze strategies associated with successful collision avoidance, relevant gaze-related parameters were analyzed as a function of participant group (HVFD_A_ patients, HVFD_I_ patients, and normal subjects) for traffic density 50 and 75%. Normal subjects and HVFD_A_ patients shared many similarities regarding their gaze patterns and differed in parameters regarded as relevant for functional compensation. In both densities, no differences were found for number of fixations, fixation duration, scan-path length, and number of gaze shifts (between the left and right side of the intersecting street). However, HVFD_A_ patients went to larger gaze eccentricities and made more fixations on cars, presumably resulting in a good estimate of the POC and successful collision avoidance. Additionally, a higher proportion of fixations and gaze eccentricity, and shorter saccades to the blind hemifield were evident for HVFD_A_ patients. Interestingly, HVFD_A_ patients displayed increased gaze eccentricity (i.e., more scanning activity) especially in the first and middle part of the approach, which indicates the importance of gaining an initial overview of the scene (see Figures 8A,B). In summary, HVFD_A_ patients displayed distinct gaze patterns characterized by an overall increased exploration, particularly toward moving objects of interest on their blind side, to adapt successfully to their visual deficit. This compensatory behavior becomes especially evident during the more demanding task, i.e., the high traffic density condition.

On the other hand, HVFD_I_ patients exhibited gaze patterns completely distinct from normal subjects. All examined gaze-related parameters (except for number of fixations and fixation duration) were significantly different between the two subgroups of patients and illustrated a decreased and unorganized visual exploration of HVFD_I_ patients (cf. Figures 8A,C). Compared to normal subjects, these differences may explain the failure of compensation in HVFD_I_ patients.

In conclusion and to bring the findings from Papageorgiou et al. (2012c) together with other studies about functional compensation (Zihl, 1995, 1999; Schuett et al., 2009; Hardiess et al., 2010), patients classified as adequate (in order to compensate for the visual field defect) are able to use different compensatory gaze strategies, which are gradually intensified as task complexity increases. Compensational gaze scanning led to a more efficient (superior) fixation pattern for HVFD_A_ patients, providing more fixations on cars than HVFD_I_ patients (and healthy controls). This strategy resulted in a better identification of the collision-relevant vehicles and successful (adequate) collision avoidance.

Besides visual field deficits, HFVD patients have been shown to suffer from brain lesions in regions commonly associated with visuo-spatial memory (Machner et al., 2009; Hardiess et al., 2010; Papageorgiou et al., 2012b). In these studies, inadequate patients were found to show larger lesions than adequate patients, especially in mesio-ventral areas of the temporal lobe (i.e., the fusiform gyrus), the inferior occipital lobe, and the para-hippocampal gyrus. In some HVFD_I_ patients, the right posterior parietal cortex or left parietal regions were affected. Temporal regions belong to the ventral processing visual stream, thought to be involved in objects recognition (Ungerleider and Mishkin, 1982) and may also play a role in the control of attention (Goodale and Milner, 1996; Ungerleider and Pasternak, 2004). Disturbance of attentional modulation within the ventral processing stream and damage of its connections with temporal lobe areas and the prefrontal cortex might be impair visuo-spatial memory. Since the para-hippocampal gyrus serves as the main pathway between the hippocampus and cortical association areas, its damage can lead to many cognitive deficits including a decline in memory representation.

Collision avoidance is a cognitively complex task, involving processes such as oculomotor adaptation, speed estimation, scanning and selection of cars with high POC, storage in visual working memory and visuo-motor calibration (Lee, 1976; Simpson et al., 2003). Consequently, together with gaze compensation, the role and function of working memory must be considered when investigating adequate street crossing in hemianopes. Working memory may contribute in three ways (Martin et al., 2007; Machner et al., 2009; Hardiess et al., 2010): (1) supporting visual perception, (2) providing memory-guided saccades, and (3) representing cars and performing calculation of their POC.

Due to the visual field deficit, HFVD patients need to invest additional effort in visual search of the scene in order to select the task-relevant obstacles (cars with high POC), which will be represented in working memory. Insufficient visual exploration or reduced working memory capacity lead to inadequate compensation. A further compensatory option for HVFD_A_ patients is to use their intact working memory in order to perform memory-guided saccades (Martin et al., 2007; Hardiess et al., 2010; Papageorgiou et al., 2012c), particularly toward the blind hemifield, where no visual input is available. By shifting their gaze to remembered coordinates of the visual scenery in a goal-oriented manner, they are able to save time and avoid unnecessary visual search. In contrast, in HVFD_I_ patients, working memory capacity seems to be reduced, forcing them to devote a high proportion of their fixations on the intersection in order to create an adequate spatial representation of stationary elements at the cost of moving cars. Consequently, decreased gaze activity and reduced working memory availability result in their inability to solve the task.

## Summary and conclusion

Visually controlled collision detection and avoidance in the traffic intersection task requires contributions from perceptual, visuo-motor, and memory components. Studies with normal subjects indicate that gaze movement patterns are task specific in that attention is directed to cars with high POC. Variations of gaze behavior are not predictive of task performance in different subjects or in different trials. It therefore seems likely that the working memory contributions such as judging POC by anticipation of car trajectories, or keeping track of intercepting cars are the major sources of inter-individual variation. This hypothesis is confirmed by the studies with hemianopic patients who can be divided into two groups. In an adequately performing subgroup, memory contribution seems to be intact and visual field loss can be compensated for by additional gaze movements. In an inadequately performing subgroup, such compensation is not possible, presumably due to problems with the required support from spatial memory.

With the data reported here, we expand the models proposed for collision avoidance regarding multiple-object events and gaze movements. For the purpose of selecting relevant objects and estimating their actual POC, bottom-up (pre-attentive) together with top-down (cognitive) processes must be considered. The interaction of both processes implements a representation of objects based on their POC within working memory.

### Conflict of interest statement

The authors declare that the research was conducted in the absence of any commercial or financial relationships that could be construed as a potential conflict of interest.
